# The carnivorous rainbow plant *Byblis filifolia* Planch. secretes digestive enzymes in response to prey capture independently of jasmonates

**DOI:** 10.1111/plb.70029

**Published:** 2025-05-19

**Authors:** A. Pavlovič, T. Jílková, I. Chamrád, R. Lenobel, O. Vrobel, P. Tarkowski

**Affiliations:** ^1^ Department of Biophysics, Faculty of Science Palacký University in Olomouc Olomouc Czech Republic; ^2^ Laboratory of Growth Regulators, Faculty of Science Palacký University in Olomouc and Institute of Experimental Botany of the Czech Academy of Sciences Olomouc Czech Republic; ^3^ Czech Advanced Technology and Research Institute (CATRIN) Palacký University in Olomouc Olomouc Czech Republic; ^4^ Czech Agrifood Research Center, Genetic Resources of Vegetables and Special Crops Olomouc Czech Republic

**Keywords:** *Byblis*, Carnivorous plant, digestive enzyme, *Drosera*, jasmonic acid, phytohormones, sundew

## Abstract

- Carnivorous plants from the order Caryophyllales co‐opted plant phytohormones from a group of jasmonates to regulate digestive enzyme activity. However, not all genera of carnivorous plants have been thoroughly explored, and the digestive physiology of Australian carnivorous rainbow plants of the genus *Byblis* (order Lamiales) is poorly understood.
- Here, we investigated the composition of digestive enzymes in the secreted fluid of *Byblis filifolia* using LC/MS, measured enzyme activity, and analysed tissue phytohormone levels after experimental feeding with fruit flies and coronatine application.
- Several hydrolytic digestive enzymes were identified in the secreted digestive fluid, the levels of which clearly increased in the presence of insect prey. However, in contrast to the sundew *Drosera capensis*, endogenous jasmonates do not accumulate, and coronatine, a molecular mimic of jasmonates, is unable to trigger enzyme secretion.
- Our results showed that *B. filifolia* is fully carnivorous, with its own digestive enzyme repertoire. However, in contrast to carnivorous genera from the Caryophyllales order, these enzymes are not regulated by jasmonates. This indicates that jasmonates have not been repeatedly co‐opted to regulate digestive enzyme activity during the evolution of carnivorous plants.

## Graphical abstract

Secretion of digestive enzymes in response to prey capture in carnivorous plants with sticky traps is regulated by jasmonates in *Drosera capensis* but differently by unknown factor in *Byblis filifolia*.

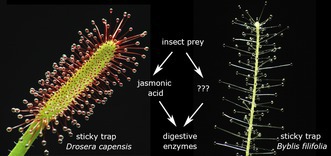

## INTRODUCTION

Carnivorous plants have evolved in nutrient‐poor, sunny, and wet habitats at least 11 times independently, and are thus an example of convergent evolution (Givnish *et al*. 1984, 2018; Adamec *et al*. 2021). To be considered carnivorous, plants must fulfil several requirements, such as specialized traps must be used to catch and kill prey, which must be digested and nutrients absorbed and used for plant growth and development (Adamec *et al*. 2021). Carnivorous plants transform their leaves into functional traps for this purpose. Five different trapping strategy can be distinguished in carnivorous plants: (i) adhesive (‘flypaper’) traps with a sticky glandular surface (e.g. *Drosera*, *Pinguicula*, *Byblis*); (ii) pitfall traps forming a pitcher or small tank (e.g. *Nepenthes*, *Sarracenia*, *Cephalotus*); (iii) mobile snap‐traps with rapidly closing trap (e.g. *Aldrovanda*, *Dionaea*); (iv) suction (‘bladder’) traps actively forming negative pressure inside (e.g. *Utricularia*); and (v) specialized eel (‘lobster‐pot’ and ‘cork‐screw’) traps formed by screwed, tubular leaves with a narrow cavity lined with hairs (e.g. *Genlisea*, Adamec *et al*. 2021).

*Byblis* is a small genus of carnivorous plant with adhesive (‘flypaper’) traps in the order Lamiales, currently consisting of eight recognized species. Two perennial species (*B. gigantea* and *B. lamellata*) grow in Western Australia, and six annual species (*B. aquatica*, *B. filifolia*, *B. guehoi*, *B. liniflora*, *B. pilbarana*, and *B. rorida*) grow from Northern Australia to the island of New Guinea (Lowrie & Conran 1998; Conran *et al*. 2002a, 2002b; Fukushima *et al*. 2011; Lowrie 2013). All species produce filiform leaves covered in two gland types: stalked and sessile glands. Regarding carnivory, the genus *Byblis* is highly understudied compared to other carnivorous plant genera because of limited plant availability, the annual character of many species, and difficult cultivation and propagation. The genus *Byblis* has been considered a passive flypaper for over two centuries, but recently it was found that stalked glands are able to perform motion responses to chemical stimulation. The movement consists of a sequence of twisting and kinking motions powered by water displacement processes from the gland stalk to the basal cells, and the traps are now considered as active flypaper (Allan 2019a; Poppinga *et al*. 2022).

The ability to produce digestive enzymes has been questioned several times in the genus *Byblis*. Bruce (1905) was the first to observe apparent digestion of albumen. Subsequently, Lloyd (1942) was unable to demonstrate fibrin digestion. Hartmeyer (1997a) also concluded that *B. liniflora* could not produce digestive proteases because the plant did not digest the gelatin layer of the strips of photographic film. However, more than a century later since Bruce (1905) initial experiments, Takahashi *et al*. (2009) documented the ability of *B. liniflora* to digest proteins. Later, by extending the usual test period using strips of photographic film, Allan (2019b) and Hartmeyer & Hartmeyer (2019) obtained positive results for *B. gigantea*, *B. filifolia*, and *B. liniflora*. Li *et al*. (2023) documented the division of labor between glands, where the sessile glands are the major structures that secrete digestive enzymes, and the stalked glands serve mainly for prey capture. Because of the many uncertainties regarding the secretion of digestive enzymes, the genus *Byblis* has been suspected to rely on digestive mutualism with fungi or *Setocoris* bugs, similar to *Roridula* plants (Ellis & Midgley 1996; Hartmeyer 1997b; Anderson & Midgley 2003; Cross *et al*. 2018). However, the genus *Roridula* is now considered carnivorous, despite its inability to produce its own digestive enzymes. Instead, *Roridula* hosts bugs of the genus *Pameridea*, which prey on the trapped insects. Bugs deposit their faeces on the leaves and the plant takes up nutrients from the droppings (Ellis & Midgley 1996; Hartmeyer 1997b; Anderson & Midgley 2003; Plachno *et al*. 2009). Using the stable isotope technique, Skates (2021) clearly demonstrated an uptake of N from insect prey and found that *Byblis* does not obligate *Setocoris* to assist with digestion and assimilation of captured invertebrate prey, suggesting that the plants have to digest insect prey using their own digestive enzymes. She also found that annual species of *Byblis* gained as much N from prey as morphologically similar *Drosera* species, indicating a clear nutritional benefit from carnivory (Skates 2021).

Because our knowledge of digestive processes in the genus *Byblis* is still very fragmentary and relies more on observations than sophisticated analytical methods, we employed LC–MS analysis to address the following questions: (i) What is the enzyme composition of the digestive fluid in the genus *Byblis*; (ii) is enzyme activity induced by signals from the captured prey; and (iii) is the jasmonic acid (JA) signalling pathway involved in the regulation of digestive enzyme activity? Our study clearly shows that *B. filifolia* is a truly carnivorous plant that produces digestive enzymes upregulated by insect prey but that are not regulated by jasmonates.

## MATERIALS AND METHODS

### Plant cultivation and experimental setup

**Fig. 1:**
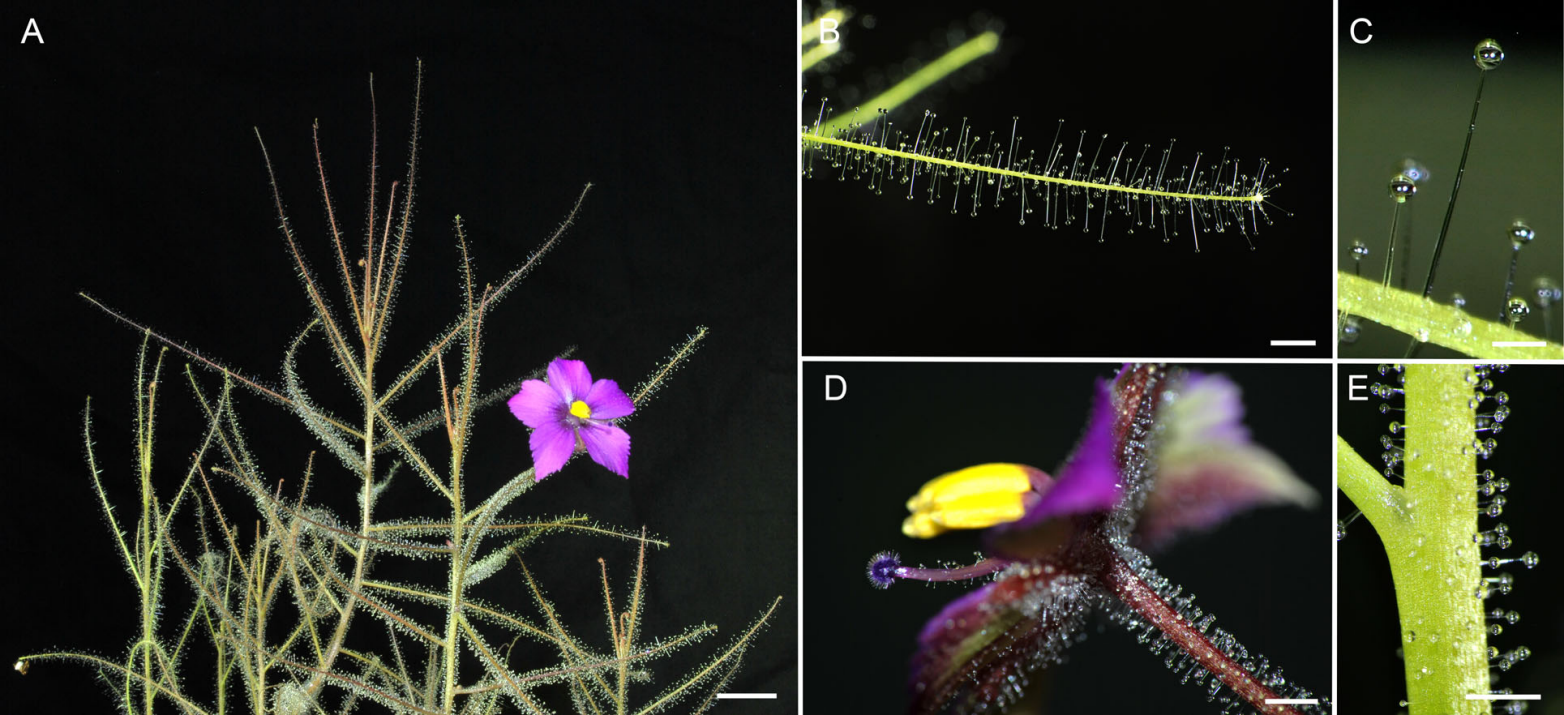
Experimental plant material *Byblis filifolia*. (A) Whole view of experimental plant, scale bar = 1 cm, (B) Detailed view of apex of sticky leaf, scale bar = 2 mm, (C) Detailed view of leaf stalked glands, scale bar = 0.5 mm, (D) Detailed view of flower with sticky trichomes on sepals, stigma and pedicel, scale bar = 1 mm. (E) Detailed view of sticky stem, scale bar = 2 mm.

*Byblis filifolia* Planch. (Fig. 1) plants were cultivated from seeds kindly provided by Siegfried Hartmeyer (Weil am Rhein, Germany). They were sown on peat moss: silica sand (1:1) substrate in plastic pots (7 × 7 × 7 cm) and placed in a tray filled with distilled water to a depth of 1–2 cm. Germination was further stimulated by placing the tray with pots into a growth chamber at elevated temperature (30–35°C), 100% relative air humidity, and 100 μmol m^−2^ s^−1^ photosynthetic active radiation (PAR) for 2 weeks. The seedlings were then cultivated on the sill of a south‐facing window exposed to full sunlight. Although the seeds germinated well, plant mortality was high in the first month after sowing. Cape sundew plants (*Drosera capensis* L.) cultivated from the seeds under the same conditions were used as positive controls.

For the feeding experiments, the apical part of subadult *B. filifolia* plants (5 months old, 15–20 cm high) was fed 30 fruit flies (*Drosophila melanogaster*) with a density of 5–10 flies per leaf. Flies were cultured from eggs in a carbohydrate‐rich medium. For analysis of digestive fluid composition and enzyme activity, another group of plants was sprayed with 100 μM coronatine in 0.01% Tween 20 (Sigma‐Aldrich, St. Louis, MO, USA) or only with 0.01% Tween 20 (control group). The feeding response and potency of the coronatine treatment were also tested on leaves of sundew plants (*D. capensis*), which strongly folded within a few hours in response to coronatine application (Nakamura *et al*. 2013). Five control, five fed, and five coronatine‐treated similar‐sized leaves were cut off using scissors after 24 h, and the leaves were submerged one by one in 4 mL 50 mM sodium acetate buffer solution (pH 5.0), each for 3 min to collect exudates (Matušíková *et al*. 2005; Krausko *et al*. 2017). Each leaf was repeatedly emerged and immersed in the buffer solution until all the viscous exudate drained from the leaf. To analyse the tissue phytohormone levels, we included another group of plants whose leaves were wounded several times with tweezers. The apical parts of the control, fed, and wounded plants were collected after 2 h and immediately frozen in liquid nitrogen. These time points were chosen based on previous experiments with carnivorous plants, which used sticky traps and because JA culminated at this time point (Nakamura *et al*. 2013; Mithöfer *et al*. 2014; Krausko *et al*. 2017; Kocáb *et al*. 2020; Pavlovič *et al*. 2024). The sundew plant *D. capensis* was used as a positive control at exactly the same time points.

### Enzyme activity measurements

Freshly collected digestive fluids were used to measure enzyme activity. The proteolytic activity of the digestive fluid was determined by incubating 150 μL of the collected sample with 75 μL 2% (w/v) bovine serum albumin in 200 mM glycine–HCl (pH 3.0) at 37°C for 4 h. The reaction was stopped by the addition of 200 μL 10% (w/v) trichloroacetic acid (TCA). Samples were incubated on ice for 10 min and then centrifuged at 20,000×*g* for 10 min at 4°C. The supernatants from the samples were pipetted into a UV transparent 96‐well microplate. The amount of released non‐TCA‐precipitable peptides was used as a measure of proteolytic activity, which was determined by comparing the absorbance of the supernatant at 280 nm with that of a blank sample using a SynergyMx microplate reader (BioTek Instruments, Winooski, VT, USA). One unit of proteolytic activity was defined as an increase of 0.001 min^−1^ in the absorbance at 280 nm (Matušíková *et al*. 2005).

To measure phosphatase activity, 5 mM 4‐nitrophenyl phosphate (Sigma‐Aldrich) in 50 mM acetate buffer (pH 5.0) was used as substrate. A 150 μL sample of the collected digestive fluid was added to 400 μL acetate buffer and mixed with 400 μL substrate. As a control, 400 μL substrate solution was added to 550 μL buffer. Mixed samples were incubated at 25°C for 2 h. Thereafter, 160 μL 1.0 M NaOH was added to terminate the reaction. Absorbance was measured at 410 nm using a Specord 250 Plus double‐beam spectrophotometer (Analytik Jena, Germany).

### Western blotting

The digestive fluid collected for the enzyme assays was subjected to Western blotting. The samples were heated and denatured for 30 min at 70°C and mixed with modified Laemmli sample buffer to a final concentration of 50 mM Tris–HCl (pH 6.8), 2% SDS, 10% glycerol, 1% β‐mercaptoethanol, 12.5 mM EDTA, and 0.02% bromophenol blue. The same volume (25 μL) of samples was electrophoresed in 10% (v/v) SDS polyacrylamide gel (Schägger 2006). Proteins in the gels were either visualized by silver staining (ProteoSilver; Sigma‐Aldrich) or transferred from the gel to a nitrocellulose membrane (Bio‐Rad) using a Trans‐Blot SD Semi‐Dry Electrophoretic Transfer Cell (Bio‐Rad, Hercules, CA, USA). After blocking in TBS‐T containing 5% BSA overnight, the membranes were incubated with the primary antibody for 1 h at room temperature and then, after washing, the membranes were incubated with the secondary antibody (goat anti‐rabbit IgG (H + L) horseradish peroxidase conjugate (Bio‐Rad)). Blots were visualized and chemiluminescence quantified using an Amersham Imager 600 gel scanner (GE Healthcare Life Sciences, Tokyo, Japan).

### Phytohormone analyses

Analysis of phytohormones was carried out as previously described in Pavlovič *et al*. (2023, 2024). The collected plant tissues were frozen in liquid nitrogen and ground using a mortar and pestle. The 25 mg samples were extracted with 1 mL ice cold 50% acetonitrile (ACN) containing a mixture of stable isotope‐labelled standards. Unlabelled (SA, JA‐Ile, ABA) and labelled standards (D4‐SA, D6‐ABA) were purchased from OlChemIm (Olomouc, Czech Republic); JA and D5‐JA were purchased from Merck (Darmstadt, Germany). The extraction was performed with assistance of an ice‐cold ultrasonic bath for 30 min. After centrifugation (20,000×*g*, 15 min, 4°C), samples were purified using solid phase extraction (SPE). Waters (Milford, MA, USA) Oasis™ HLB columns (30 mg, 1 mL cartridge) were activated by 1 mL MeOH and equilibrated by 1 mL H_2_O and 1 mL 50% ACN. During sample loading, the flow‐through fraction was collected and pooled with the fraction from a single‐washing step of 1 mL 30% ACN. Collected fractions were evaporated under a vacuum. If necessary, the dried samples were stored at −20°C prior to analysis. For analysis, samples were resuspended in 40 μL mobile phase, filtered through 0.2‐μm microspins (Ciro, Deerfield Beach, FL, USA) and analysed via liquid chromatography (positive) electrospray ionization tandem mass spectrometry (LC‐(+)ESI‐MS/MS) in the multiple reaction monitoring (MRM) mode. LC–MS/MS analysis was performed using a Nexera X2 modular liquid chromatograph system coupled to an MS 8050 triple quadrupole mass spectrometer (Shimadzu, Kyoto, Japan) via an electrospray interface. Chromatographic separation was performed using a reverse‐phase analytical column, Waters CSH™ C18, 2.1 × 150 mm, 1.7 μm. The aqueous solvent A consisted of 15 mM formic acid adjusted to a pH of 3.0 with ammonium hydroxide. Solvent B was pure ACN. Separation was achieved with gradient elution at a flow rate of 0.4 mL min^−1^ at 40°C. Then, 0–1 min 20% B and 1–11 min 80% B linear gradients followed by washing and equilibration to initial conditions for a further 7 min were applied. If possible, up to three MRM transitions (one quantitative, the others qualitative) were monitored for each analyte to ensure as much confidence as possible in the correct identification of analytes in the different plant matrices. Raw data were processed via Shimadzu software LabSolutions ver. 5.97 SP1.

### Proteomic analysis of digestive fluids

Proteins from *Byblis* digestive fluids were identified following the methodology published in Pavlovič *et al*. (2024). Briefly, the collected digestive fluid in 50 mM sodium acetate buffer solution (pH 5.0) was combined with Laemmli sample buffer in a ratio of 1:1 (v/v) and its protein component separated by SDS‐PAGE. The resolved, Coomassie‐stained proteins were digested *in‐gel* with trypsin, and the obtained peptides were desalted employing C18 Stage Tips and analysed by LC‐ESI‐MS/MS on a timsTOF PRO 2 mass spectrometer (Bruker Daltonik) connected to a nano‐flow capillary liquid chromatography system RSLCnano (Dionex, Thermo Fisher Scientific). All specific settings were identical to those used by Vlčko *et al*. (2023). MS data were processed with DataAnalysis v. 4.3 (×64, Bruker Daltonics), uploaded into the Peaks X Pro software (Bioinformatic solutions, ON, Canada; Tran *et al*. 2018) and searched against Lamiales protein database downloaded from the NCBI repository (1263276 sequences; downloaded on November 13^th^ 2023). The search settings used with Denovo, PEAKS, PEAKS PTM, and SPIDER options were as follows: enzyme – trypsin; digest mode—semispecific; fixed modification—carbamidomethylation; variable modification—acetylation (protein N‐term), oxidation of methionine, and deamidation of glutamine and asparagine; MS mass error tolerance at 50 ppm; MS/MS mass error tolerance at 0.05 Da. The obtained SPIDER search results were filtered by applying 1% and 10% FDR limits for peptides and proteins, respectively, with at least one unique peptide required for unambiguously assigned protein. Localization of the identified proteins was inspected with the use of DeepLoc 2.0 multi‐label predictor (Thumuluri *et al*. 2022), and the function of sequences missing annotation was assessed by conserved domain analysis performed with the Conserved Domain Database (Wang *et al*. 2023). Multiple sequence alignment of S8 subtilisin homologues was performed using T‐Coffee web server (Di Tommaso *et al*. 2011). The original raw MS data, together with the respective protein identification data with all relevant characteristics provided by the Peaks X Pro software, have been deposited to the ProteomeXchange Consortium (http://proteomecentral.proteomexchange.org) via the PRIDE partner repository (Vizcaíno *et al*. 2013) with the dataset identifier PXD060459. and are summarized in Table S1.

### Statistical analysis

Before statistical analyses, the data were tested for normality (Shapiro–Wilk test) and homogeneity of variance (Brown–Forsythe test). If normality and homogeneity were fulfilled, parametric one‐way ANOVA with Tukey's post‐hoc test was used for multiple comparisons. If not, non‐parametric Kruskal–Wallis test with Dunn's post‐hoc test was used instead. For two data sets, Welch's test or Mann–Whitney *U* test were used for parametric and non‐parametric data, respectively (Origin 8.5.1, Northampton, MA, USA).

## RESULTS

### Byblis upregulates enzyme activity in response to feeding but not to coronatine application

**Fig. 2:**
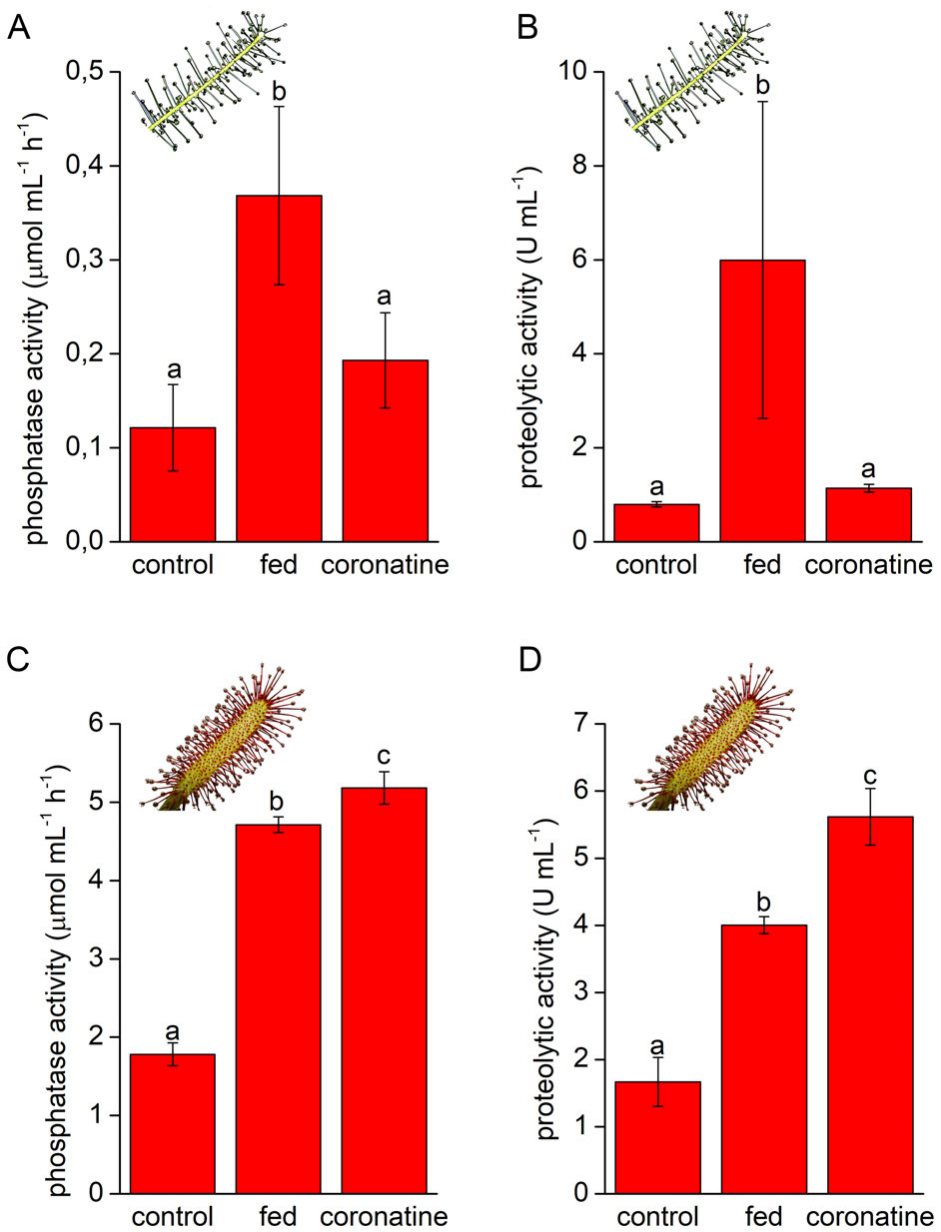
Enzyme activity measurements in *Byblis filifolia* (A, B) and *Drosera capensis* (C, D) in response to feeding and coronatine application. (A, C) Phosphatase activity; (B, D) Proteolytic activity. Data are means ± SD, *n* = 4–6. Different letters denote significant differences at *P* < 0.05 (ANOVA, Tukey's test).

**Fig. 3:**
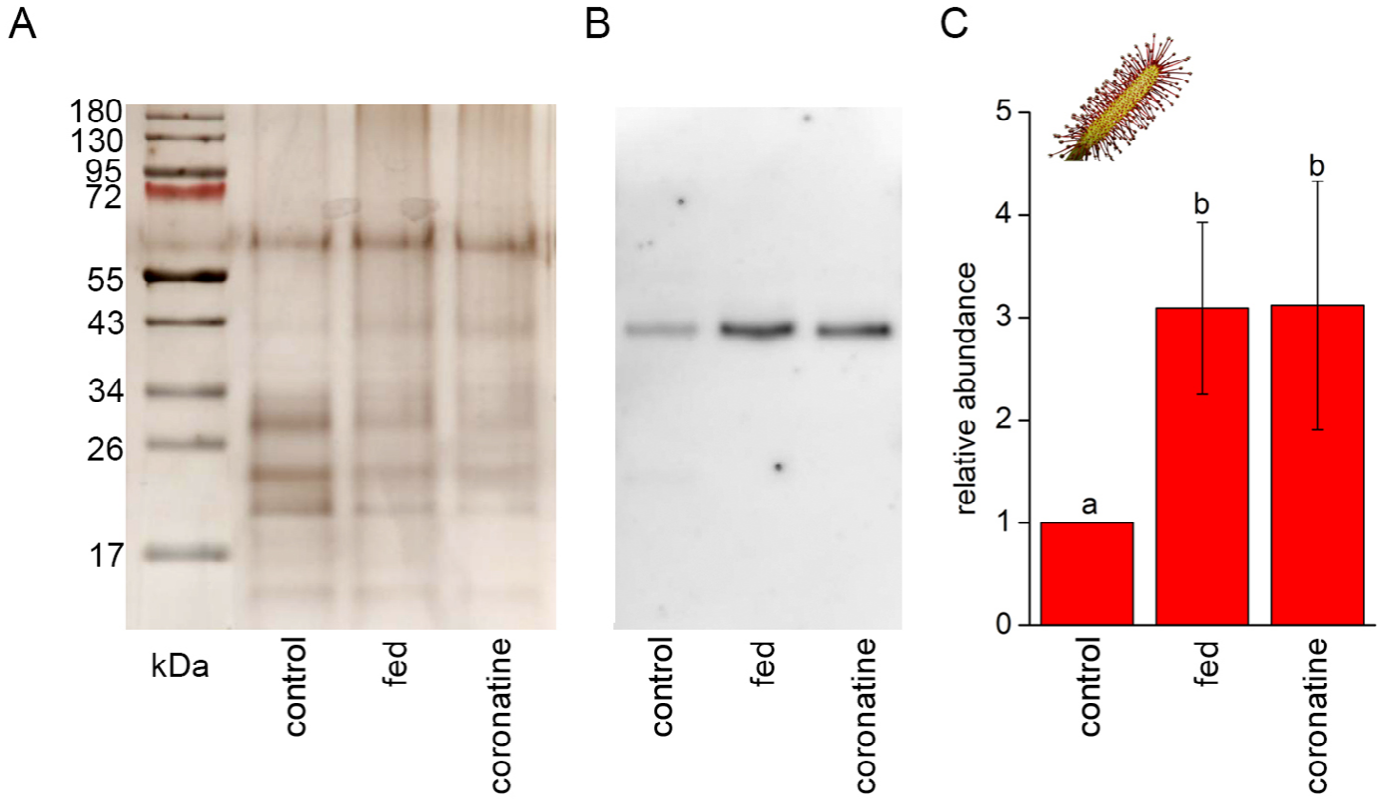
Immunodetection of aspartic protease in *Drosera capensis* exudate in response to feeding and coronatine application. (A) Separated proteins in 10% (v/v) SDS‐PAGE stained with silver, (B) Immunodetection of aspartic protease, (C) Quantification of chemiluminescence signal intensity. The same volumes (25 μL) of samples were loaded. Mean ± SD, *n* = 4. Different letters denote significant differences at *P* < 0.05 (ANOVA, Tukey's test).

Application of live fruit flies significantly increased phosphatase and proteolytic activity in sticky exudates of *B. filifolia* and *D. capensis* plants (Fig. 2). Despite the high variation in the sample replicates of the fed *B. filifolia* plants, the differences were significant; however, different responses were observed in response to 100 μM coronatine application. Although neither enzyme activity was significantly upregulated in the exudate of *B. filifolia* (Fig. 2A,B), it was clearly upregulated in *D. capensis* plants (Fig. 2C,D). For quantification of digestive enzymes, we used four antibodies from our previous studies focused on *Dionaea* and *Nepenthes* plants (Pavlovič *et al*. 2017; Saganová *et al*. 2018) but we were successful only in immunodetection of aspartic protease in *D. capensis* plants. The abundance of aspartic protease in the digestive fluid correlated with proteolytic activity, with the highest abundance found in the fed and coronatine‐treated plants (Fig. 3). No antibody was immunoreactive in the exudates of *B. filifolia* plants, indicating that either the enzyme was not present in the digestive fluid or the epitope sequence was different, and the antibody was unable to bind to the target protein. Therefore, we proceeded to LC/MS analysis of the digestive fluids in *B. filifolia*.

### Identification of digestive enzymes

Proteomic analysis of *B. filifolia* digestive fluid provided an unambiguous identification of 39 proteins (Table S1). These proteins are of plant origin because their sequences show strong homology with known plant proteins in the Lamiales database used for identification. Eight were predicted to have extracellular localization and enzyme activity typical of plant digestive fluids, such as esterase/lipase, phosphatase, chitinase, and protease (Table 1). Interestingly, the latter belongs to the S8 subtilisin‐like serine protease family with an Asp/His/Ser catalytic triad (Antão & Malcata 2005) rather than to the S10 carboxypeptidase family found in other carnivorous plant species. More specifically, a total of five different S8 subtilisin sequences with homology ranging from 54.85% to 96.2% were detected, each with at least one unique peptide (Figure S1). As suggested by the semi‐quantitative spectral counts, most of the proteins were upregulated only in response to feeding and not to coronatine application (see PSMs values in Table 1). However, despite these novel findings, the obtained dataset is still likely to be incomplete because our identification strategy is based on the protein homology searches (Hatano & Hamada, 2012; Pavlovič *et al*. 2024) and proteins with low sequence similarity to known Lamiales sequences may have remained undetected.

**Table 1:** Protein component of *Byblis filifolia* digestive fluid characterized by mass spectrometry.

protein accession^a^	protein name	organism	peptides/SC^b^	PSMs^c^	function^d^	localization^e^
2127213838	Hypothetical protein C2S51_000637	*Perilla frutescens*	9/13	3/9/3	S8 family subtilisin‐like serine protease	Extracellular
2127213839	Hypothetical protein C2S51_000638	*Perilla frutescens*	7/9	5/12/6	S8 family subtilisin‐like serine protease	Extracellular
2127214961	Hypothetical protein C2S51_001760	*Perilla frutescens*	5/7	1/7/3	S8 family subtilisin‐like serine protease	Extracellular
1912748405	Subtilisin‐like protease sdd1	*Phtheirospermum japonicum*	3/6	1/4/1	S8 family subtilisin‐like serine protease	Extracellular
2320562803	GDSL esterase/lipase At1g29670‐like	*Andrographis paniculata*	2/5	4/4/2	SGNH/GDSL hydrolase family protein	Extracellular
1746580330	Purple acid phosphatase 24	*Striga asiatica*	2/7	0/1/0	Purple acid phosphatase	Extracellular
2236153894	Subtilisin‐like protease	*Salvia hispanica*	2/1	2/8/2	S8 family subtilisin‐like serine protease	Extracellular
2506127979	Unnamed protein product	*Fraxinus pennsylvanica*	2/7	2/1/0	Family 18 glycoside hydrolase/chitinase	Cytoplasm, Extracellular

^a^ NCBI database accession.

^b^ SC—sequence coverage in %.

^c^ PSMs—peptide‐spectrum matches for control, feeding experiment and coronatine treatment, respectively.

^d^ Conserved Domain Database functional annotation.

^e^ Cellular localization predicted with DeepLoc 2.0.

### Byblis does not accumulate jasmonates in response to feeding

**Fig. 4:**
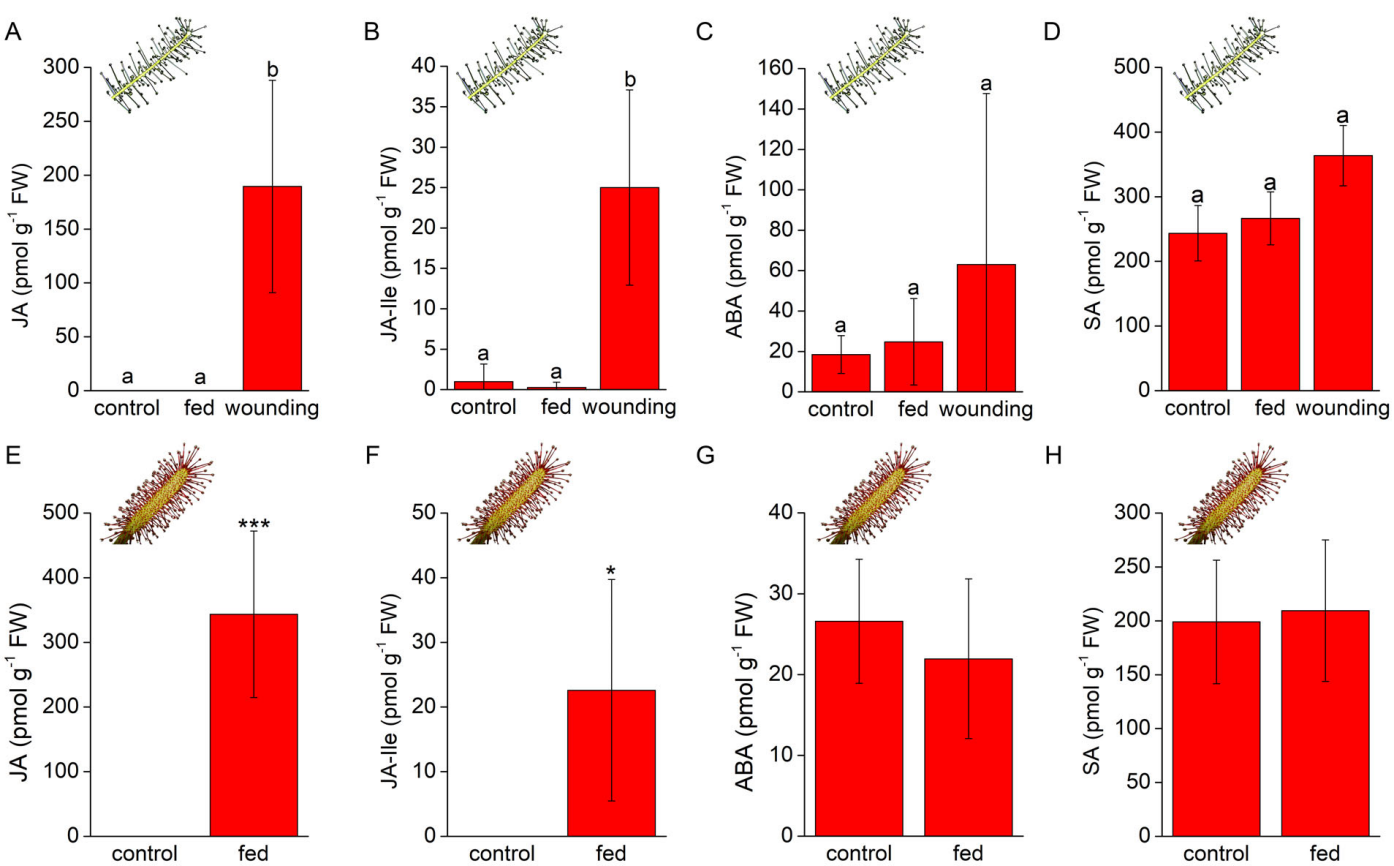
Tissue phytohormone level in *Byblis filifolia* (A–D) and *Drosera capensis* (E–H) in response to feeding and coronatine application. (A, E) jasmonic acid; (B, F) isoleucine conjugate of jasmonic acid (JA‐Ile); (C, G) abscisic acid; (D, H) salicylic acid. Mean ± SD, *n* = 4–7. (A–D) Different letters denote significant differences at *P* < 0.05 (ANOVA, Tukey's test or Kruskal‐Wallis, Dunn's test), (E–H) asterisks denote significant differences at *P* < 0.05 (*) and *P* < 0.001 (***) (Welch's *t*‐test).

Phytohormone analysis did not show elevated endogenous levels of JA or its isoleucine conjugate (JA‐Ile) in response to feeding in *B. filifolia* (Fig. 4A,B). Other stress‐related hormones such as abscisic acid (ABA) and salicylic acid (SA) did not accumulate (Fig. 4C,D). As a positive control, wounded *B. filifolia* plants showed significantly increased levels of JA and JA‐Ile despite high variation in individual replicates (Fig. 4A,B), which is typical for any wounded plant. The ABA and SA contents were slightly, but not significantly, higher in wounded plants. Sundew *D. capensis* clearly and significantly accumulated JA and JA‐Ile (Fig. 4E,F), but not ABA or SA, in response to feeding (Fig. 4G,H).

## DISCUSSION

The ability of carnivorous plants of the genus *Byblis* to produce digestive enzymes has been enigmatic due to the scarcity of these carnivorous plants in private and scientific collections for rigorous experimental investigations because of cultivation and propagation difficulties. Many experiments have been conducted by carnivorous plant enthusiasts using simple methods for the detection of proteolytic enzymes, such as the substrate film method, with negative (Hartmeyer 1997a) and positive (Hartmeyer & Hartmeyer 2019; Allan 2019b) results. Previously, the genus *Byblis* was suspected to rely more on digestive mutualism than on the production of secreted digestive enzymes (Juniper *et al*. 1989; Ellis & Midgley 1996; Anderson & Midgley 2003; Cross *et al*. 2018). Here, using LC–MS analysis, we clearly dispelled these doubts, and identified several groups of digestive enzymes similar to those described previously in different genera of carnivorous plants. Serine proteases, purple acid phosphatase, GDSL lipase, and chitinase were identified. All these enzymes have been identified in carnivorous plants within the order Caryophyllales (*Drosera*, *Dionaea*, *Nepenthes*, *Drosophyllum*), Ericales (*Sarracenia*), Lamiales (*Pinguicula*), or Oxalidales (*Cephalotus*) (Hatano & Hamada 2012; Schulze *et al*. 2012; Lee *et al*. 2016; Rottloff *et al*. 2016; Fukushima *et al*. 2017; Krausko *et al*. 2017; Kocáb *et al*., 2020; Pavlovič *et al*. 2024). This provides further evidence that carnivorous plants from different evolutionary lineages have co‐opted similar digestive enzymes (Fukushima *et al*. 2017). However, the presence of the S8 group of serine protease called subtilisins has not been previously detected in other genera of carnivorous plants, except for one study on *Nepenthes rafflesiana* (Zulkapli *et al*. 2021). Subtilisins have been implicated in diverse processes, such as pathogen defence, stomatal and leaf development, lateral root emergence, xylem differentiation, stress responses, programmed cell death, nodulation and nitrogen fixation, microsporogenesis, and seed development. However, their exact functions in these pathways remain unclear (Tripathi & Sowdhamini 2006; Figueiredo *et al*. 2018). This is another example that occasionally, carnivorous plants co‐opted also some unique enzyme types, except for the well‐known subset of digestive enzymes. Another example is the presence of α‐amylase which is unique in the digestive fluid of the genus *Pinguicula* (Kocáb *et al*. 2020).

Many carnivorous plants upregulate their enzymatic activity in response to prey capture. The signals, which trigger enzyme synthesis and secretion, are usually mechanical and chemical (Pavlovič & Mithöfer 2019). The most studied are carnivorous plants from the order Caryophyllales, mainly sundew (*Drosera* sp.) and Venus flytrap (*Dionaea muscipula*), for which the sequence of events after prey capture has been well described. After the prey has been captured, mechanical stimuli from the struggling prey elicit electrical signals (i.e. action potentials) in the plant. One of the electrical signal components is calcium, whose concentration in the cytoplasm ([Ca^2+^]_cyt_) increases rapidly after the detection of the prey mechanical stimulus (Suda *et al*. 2020; Procko *et al*. 2022). Increased [Ca^2+^]_cyt_ possibly together with other cellular factors triggers the accumulation of JA‐Ile, which induces the expression of digestive enzymes. Chemical cues from insect prey (e.g. proteins and chitin) amplify the entire process (Libiaková *et al*. 2014; Bemm *et al*. 2016; Krausko *et al*. 2017; Jakšová *et al*. 2020). The whole process of digestive enzyme induction resembles plant responses to herbivore or pathogen attacks, suggesting that carnivorous plants co‐opted plant defence mechanisms for botanical carnivory (Pavlovič & Saganová 2015; Bemm *et al*. 2016). However, *Byblis* operates the digestive system differently. Although we clearly showed that *B. filifolia* increased the production of digestive enzymes in response to prey capture, similar to other carnivorous plants, it did not use jasmonates. Jasmonates did not accumulate in response to prey capture, and coronatine, an agonist of the JA‐Ile signal, was unable to trigger digestive enzyme secretion. Similar responses have been found in different genera of carnivorous plants (*Pinguicula*, *Utricularia*, *Cephalotus*, *Sarracenia*) outside Caryophyllales, indicating that jasmonate signalling has been co‐opted only once in the Caryophyllales order, which accumulate JA‐Ile in response to feeding and trigger enzyme secretion in response to coronatine treatment (Kocáb *et al*. 2020; Jakšová *et al*. 2021; Pavlovič *et al*. 2024). Considering the rapid chemonastic (but not thigmonastic) movement of stalked glands in *Byblis* (Poppinga *et al*. 2022), *B. filifolia* probably upregulates enzyme activity mainly in response to chemical stimuli from insect prey, as also occurs in other genera of carnivorous plants (e.g. *Drosera*, *Dionaea*, *Nepenthes*) (Bemm *et al*. 2016; Saganová *et al*. 2018; Jakšová *et al*. 2020; Pavlovič *et al*. 2023). However, the endogenous signals that transform this information into a physiological response remain unclear. We can only speculate about the involvement of Ca^2+^ ions, as is known for trichomes and tentacles in non‐carnivorous and carnivorous plants, respectively (Matsumura *et al*. 2022; Pavlovič 2022; Procko *et al*. 2022), and/or rapid osmotic changes in digestive glands triggered by rapid prey‐induced Cl^−^ efflux followed by water/digestive fluid flows, as documented in the related *Pinguicula* (Heslop‐Harrison & Heslop‐Harrison 1980; Kocáb *et al*., 2020). Indeed, water transport is involved in the function of *Byblis* stalked glands (Poppinga *et al*. 2022). The presence of the DNA demethylase DEMETER‐like protein in the digestive fluid with higher spectral counts in fed plants (Table S1) may also indicate that promoter demethylation of the genes encoding digestive enzymes plays a role in this process, as has already been documented in carnivorous plants (Nishimura *et al*. 2013; Arai *et al*. 2021). These are interesting topics for further investigation.

Here, we clearly showed that plants of the genus *Byblis* are fully carnivorous with their own digestive enzyme repertoire. The secretion of these enzymes is strongly enhanced by prey capture, but in contrast to carnivorous plants within the order Caryophyllales, not regulated by jasmonates. Although we analysed only one of eight recognized species of *Byblis*, the species are very similar to each other, the genus was divided into only two species for a long time, namely *B. gigantea* and *B. liniflora*, and only recently split into eight separate species (Lowrie & Conran 1998; Conran *et al*. 2002b). Therefore, we believe that our findings on the digestive physiology of *B. filifolia* can be applied to the entire genus *Byblis* or at least to all annual species. Because *B. filifolia* is a non‐model plant species, our current study also has several limitations, such as the lack of a species‐specific sequence database for protein identification, unknown genome sequences for gene expression analysis of corresponding enzymes, and a low number of plants owing to difficult cultivation and propagation, which prevented us from conducting in‐depth experiments. In this context, sequencing the *Byblis* genome will provide a crucial resource for more comprehensive analyses in future studies.

## AUTHOR CONTRIBUTIONS

AP designed the study and wrote the manuscript; AP and TJ measured enzyme activities, did SDS‐PAGE and Western blots; TJ, IC, and RL performed protein identification; TJ, OV, and PT performed phytohormone analyses. All the authors read and edited the manuscript.

## CONFLICT OF INTEREST

The authors declare no conflicts of interest.

## Supporting information


**Table S1.** Protein identification characteristics supplemented with localization predictions and functional annotation.

**Figure S1.** Multiple sequence alignment of the identified S8 subtilisin homologues; the detected peptides are highlighted.

## Data Availability

The MS proteomics data have been deposited to the ProteomeXchange Consortium (http://proteomecentral.proteomexchange.org) via the PRIDE partner repository with the dataset identifier PXD060459.
