# Supplementary material for: The carnivorous rainbow plant Byblis filifolia Planch. secretes digestive enzymes in response to prey capture independently of jasmonates
Source: Plant Biol (Stuttg). 2025 May 19;27(4):549–57. doi: 10.1111/plb.70029 (PMC12096042; doi:10.1111/plb.70029)
Supplement: Supplementary file 2 — Figure S1. Multiple sequence alignment of the identified S8 subtilisin homologues; the detected peptides are highlighted. [file PLB-27-549-s001.pdf]

```
CLUSTAL W (1.83) multiple sequence alignment

1912748405      --------------------------------------------------
2127213838      MTLVSFTITIVCIVSITRLPKFATEIPNKNTLQTYIVHVEGPDDQQ-HES
2127213839      ML------------SFITCPAFSANTQKQTSLPTYIVHVEASHAQLSSES
2127214961      ML------------SFITCPAVSANTQEQTSLPTYIVHVEASHAQLSSES
2236153894      MASFS-KITQILLISLIFHSTITQAIDEIDDLETYIVHVDPSDTSL----


1912748405      --------------------------------------------------
2127213838      QDLQSWYRSFLPAATTSSSGEDEARLVYSYRHVFKGFAAKLSTDEVNEME
2127213839      SQLENWYRSFLPATTTASADEGEARMVYSYRSVFKGFAARLSADEVKEMA
2127214961      SQLESWYRSFLPATTTASADEGEARMVYSYRSVFKGFAARLSADEVKEMA
2236153894      EELESWYRSFLGNS-------NPSRIVHTYKHVIKGFAARLTPDEVAIIQ


1912748405      ------------MYQLSTTRSPSFLGLHQGTGLWNQSNGGKGVIIGVIDT
2127213838      KKEGFISARPQKQLSLHTTHSPNFLGLNRNVGFWKDSNYGKDVIIGVMDT
2127213839      KKEGFISARPEKILSLHTTHSPNFLGLNQNMGLWKDSNYGKGVIIGVLDT
2127214961      KKEGFISARPEKILSLHTTHSPNFLGLNQNMGLWKDSNYGKGVIIGVLDT
2236153894      KYDGFLHARPQRQLPLHTTHTPNFLGLHRETGLWEASNYGEGVIIGVLDT
                               * **::*.****::  *:*: ** *:.*****:**

1912748405      GVFPSHPSFSGANMLPPPAKWKGKCEFPNKTDCNNKLIGARLFNRAVSSA
2127213838      GVLPEHPSFSAGGMPPPPAKWKGRCQFNH-TTCNNKIIGARYFNA-----
2127213839      GILPEHPSFSDEGMPPPPAKWKGRCEFSN-TTCNNKIIGARFFGS-----
2127214961      GILPEHPSFSDEGMPPPPAKWKGRCEFMN-TTCNNKIIGARFFGS-----
2236153894      GISPDHPSFSDEGMPPPPAKWKGKCELNT-TACNNKLIGARYFTF-----
                *: *.*****  .* ********:*::   * ****:**** *

1912748405      ASILDEETVVDREGHGTHISGTAAGVFVENACFLGSACGTAVGMAPRAHL
2127213838      ----DDQSPLDDDGHGTHTASTAAGTFVGGANLLGSANGTAVGIAPLAHL
2127213839      ----DATSPFDEDGHGTHTAGTAAGSFVKGANVFGNAYGTAAGIAPMAHL
2127214961      ----DATSPFDEDGHGTHTAGTAAGSFVKGANVFGNAYGTAAGIAPMAHL
2236153894      ----GQGTSLDENGHGTHVAGTAAGSFVAGANSFRRANGTAAGIAPRAHI
                    .  : .* :***** :.**** ** .*  :  * ***.*:** **:

1912748405      AIYKVCTK--SGCSSINMLAGLDAAIEDGVDVISLSLSHQSIPFYDDHTA
2127213838      AIYKVC------CGEVDVLAGIDEAVEDGVDVISISLGLGDGDFFENSIA
2127213839      AIYRVCTP--S-CSDSRVLAGIDAAIDDGVDVLSISLGMLENSFAQDYTA
2127214961      AIYRVCTP--S-CSDSRVLAGIDAAIEDGVDVLSISLGSLTNSFAQDYIA
2236153894      AVYKVCTDTGH-CSESDVLAAMDFAIEDGVDVISLSVGLGQDPFAFNAIS
                *:*:**      *..  :**.:* *::*****:*:*:.     *  :  :

1912748405      IGTFAAVKKGIFVSCSGGNHGPFKKSIRSEAPWVLTVGASTIDRSLRATA
2127213838      IGAFSAMEKGILVSCSAGNDGPFPSSVENGAPWILTVGASTIDRKLTAKA
2127213839      IGAFSAMEKGILVSCSAGNFGPFNFSVENEAPWILTVGASTLDRKLAATA
2127214961      IGAFSAMEKGILVSCSAGNFGPFNFSIENEAPWILTVGASTLDRKLAATA
2236153894      LGAYGAVKKGVFVSCSAGNNGPSHGTLSNEAPWVLTVGASTVDRKLLAVA
                :*::.*::**::****.** **   :: . ***:*******:**.* * *

1912748405      KLGDGQEFDGESVYQQKDFPDGTMLPLVYRA----------SCA-GNTSL
2127213838      VLGNKKQFDGESAFQPKHFPQ-TLLPLVYAGMLNASDDYAPYCFYDTFNH
2127213839      VLGNNQTFDGETVFQPKDFPS-KLLPLVYAGDINTSDPYVKFCYDESLNN
```

```
2127214961      MLGNNQTFEGETVFQPKDFPS-KLLPLVYAGDINTSDPYVKFCYDESLNN
2236153894      LLGNKEGIDGETIIHISNFSQ-TQLPLIHTG----------FCISGALKG
                 **: : ::**:  : ..*.. . ***:: .          *     .

1912748405      FDVEGKVVLCDGSKDISHVDQGWEVNEAGGAAAILANREQFGFTVDANPH
2127213838      THILGKIVVCEAGG-ISATEKGEAVKSGGGAAMILINNEDRANTTIAEAH
2127213839      TNLRGKIVVCEPGG-LLGTEKGEAVKKAGGAAMILLNSQFYANTTEAEAH
2127214961      TNLRGKIVVCEPGG-LLGIEKGEAVKKAGAAAVILLNSQFYANTTEAEAQ
2236153894      INARGKIIACDRTE-EPFLSVIREARDAGAEAVIIMNDETEGDTKRLGAI
                 .  **:: *:         .    ....*. * *: * :  . *    .

1912748405      FLPATEVSYMAGQKIKAYINSTDSPTATILFGGTVIGDPLAPTVTSFSSR
2127213838      VLPATRVGYADGLKIKAYINSTAKPTATVFFQGTVIGGDQAPRVAAFSSR
2127213839      LLPATRVTYADGLKIKAYINSTSTPTATIHFQGTVIGDDRAPVVAAFSSR
2127214961      VLPATRVTYADGLTIKAYINSTSKPTATIHFRGTVIGDDRAPVVAAFSAR
2236153894      GFPTTQVGYSEGLRIKAYINSTSDPTATLSFKGTVIGDARAPIIASFSSR
                 :*:*.* *   *   ********  ****: * *****.  ** :::**:*

1912748405      GPSVQTPGLLKPDIIGPGMNILAASPFHFD-DTDTKLTFYIDSGTSMSTP
2127213838      GPNYPSPGILKPDILGPGVNILAAWPTSVEHKTNTKSTFNIISGTSMSCP
2127213839      GPNFASRGILKPDILGPGVNILAAWPTSVENNTNTKSTFNIISGTSMSCP
2127214961      GPNFSSRGILKPDILGPGVNILAAWPTSVENNTNAKSTFNIISGTSMSCP
2236153894      GPSVHSPGILKPDILGPGVNILAAWPVSIEGNAN---RFVFSSGTSMACP
                **.   : *:*****:***:***** *  .: .::    * : *****: *

1912748405      HLAGVAALLKSAHPNWSPAAIKSAIMTTAGLLNAGKTAILDEKHTPADVF
2127213838      HLSGVAALIKSAHPNWSPAAIKSAIMTTADVVNLAHNPIEDERYLPANIF
2127213839      HLSGVAALIKSAHPNWSPAAIKSAIMTTADVVNLAKNPIEDERYLPANIF
2127214961      HLSGVAALIKSAHPNWSPAAIKSAIMTTADVVNLAKNPIEDERYLPANIF
2236153894      HLSGVAALLKSAHPDWSPAAIKSAIMTTADVVNLAGSPIEDQTLKPANVY
                **:*****:*****:**************.::*  . ..* *:   **:::

1912748405      ATGAGHVDPSKAADPGLVYDLEPDDYILYLCGLGYTDEQVGKIVRKPVNC
2127213838      ATGAGHVNPSRANDPGLIYDIQPKDYIPYLCGLNYTNRQVGLFLQRRVNC
2127213839      ATGAGHVNPSRANDPGLIYDIQPKDYIPYLCGLNYTNREVGFFLQRRVNC
2127214961      ATGAGHVNPSRANDPGLIYDIQPKDYIPYLCGLNYTNREVGFFLQRRVNC
2236153894      ATGAGHVNPSRASDPGLVYDLEARDYIPYLCGLSYTDREIGMITQERVNC
                *******:**:* ****:**::. *** *****.**:.::* : :. ***

1912748405      TL--GISQGQLNYPTFSVSLG---PSQTFTRTVTNVGEPVSYYFVRIVAP
2127213838      SVKSRIPEAQLNYPSFSIVFTDKSFSQVYTRTVTNVGDPVSSYRVEISAP
2127213839      SVESSIPQGQLNYPAFTVIFKSPSISQVYTRTVTNVGEPAASYDVEVSAP
2127214961      SVESSIPQGQLNYPAFTVIFRSPSISQVYTRTVTNVGEPATSYDVEVSAP
2236153894      SQISSIPETQLNYPSFSVLFGS--DVQTYTRTVTNVGEANSSYAVKVSAP
                :     *.: *****:*:: :       *.:********:. : * *.: **

1912748405      KGVRINVKPRQLWFWRVGQKATYSVTFRRCGNMTTNDTYSQGYLQWVSRK
2127213838      RGVGVRVDPATLKFSEVNQKLQYGVRFTRLKSS-PNNTVVQGFLKWSSPN
2127213839      PGVDVRVAPASLTFPELNQKMEYEVRFNRLTSA-VNNTVVQGFLKWTSAK
2127214961      PGVDVRVAPTSLTFSELNQKMEYQVRFNRLTSA-ENNTVVQGFLKWTSAK
2236153894      PGVGVVVEPGRLDFNELRQKLTYQVTFTRDLNG-DKFNFSQGFLTWSSTR
                 ** : * *  * * .: **  * * * *   .   : .  **:* * * .

1912748405      HKVRSVISVMLTGGGGA
```

```
2127213838      HSVRSPIAVVLS-----
2127213839      HSVRSPIAVILQ-----
2127214961      HSVRSPIAVILQ-----
2236153894      YEVRSPIVAMMIDWEF-
                :.*** * .::
```
